# Wearable red–green–blue quantum dot light-emitting diode array using high-resolution intaglio transfer printing

**DOI:** 10.1038/ncomms8149

**Published:** 2015-05-14

**Authors:** Moon Kee Choi, Jiwoong Yang, Kwanghun Kang, Dong Chan Kim, Changsoon Choi, Chaneui Park, Seok Joo Kim, Sue In Chae, Tae-Ho Kim, Ji Hoon Kim, Taeghwan Hyeon, Dae-Hyeong Kim

**Affiliations:** 1 Center for Nanoparticle Research, Institute for Basic Science (IBS), Seoul 151-742, Republic of Korea; 2 School of Chemical and Biological Engineering and Institute of Chemical Processes, Seoul National University, Seoul 151-742, Republic of Korea; 3 Samsung Advanced Institute of Technology, 130, Samsung-ro, Yeongtong-gu, Suwon 443-803, Republic of Korea; 4 School of Mechanical Engineering, Pusan National University, Busan 609-735, Republic of Korea

## Abstract

Deformable full-colour light-emitting diodes with ultrafine pixels are essential for wearable electronics, which requires the conformal integration on curvilinear surface as well as retina-like high-definition displays. However, there are remaining challenges in terms of polychromatic configuration, electroluminescence efficiency and/or multidirectional deformability. Here we present ultra-thin, wearable colloidal quantum dot light-emitting diode arrays utilizing the intaglio transfer printing technique, which allows the alignment of red–green–blue pixels with high resolutions up to 2,460 pixels per inch. This technique is readily scalable and adaptable for low-voltage-driven pixelated white quantum dot light-emitting diodes and electronic tattoos, showing the best electroluminescence performance (14,000 cd m^−2^ at 7 V) among the wearable light-emitting diodes reported up to date. The device performance is stable on flat, curved and convoluted surfaces under mechanical deformations such as bending, crumpling and wrinkling. These deformable device arrays highlight new possibilities for integrating high-definition full-colour displays in wearable electronics.

## Abstract (web summary)

Deformable and high-resolution LEDs have attracted great interest for wearable electronics, but full-colour display is still challenging. Using a stamp printing technology, Choi *et al*. build ultra-thin RGB quantum dot pixel arrays with luminous efficiency of 14,000 cd m^−2^ operated at low voltage of 7 V.

## Graphical abstract

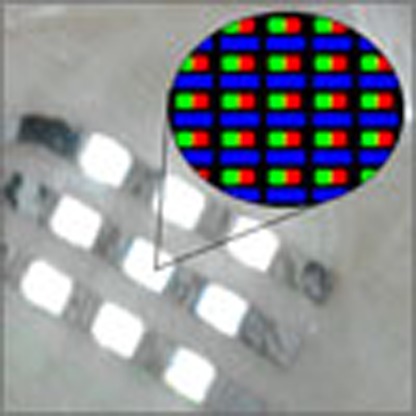

Many mobile electronic devices, including smartphones and tablets, consist of electronic and optoelectronic components, such as microprocessors, memory modules and high-resolution information displays. More advanced systems provide the higher mobility by shifting platforms from rigid/planar to wearable/deformable ones. Recently, significant progresses have been made in flexible and stretchable electronics^1^,^2^,^3^,^4^. However, the deformable, high-resolution full-colour light-emitting diode (LED) array, which is used as input/output terminals in wearable electronic systems, is a daunting goal^5^,^6^,^7^,^8^. Although previous reports showed great breakthroughs, such as flexible and/or stretchable inorganic LEDs^9^, polymer LEDs^10^,^11^,^12^ and organic LEDs^13^,^14^,^15^, practical challenges (for example, full-colour display, luminous efficiency and ultra-thin thickness) still remain.

Among various light-emitting devices, colloidal quantum dot LEDs (QLEDs) have attracted great attention as next-generation displays based on electroluminescence (EL)^16^,^17^,^18^,^19^,^20^,^21^,^22^,^23^. Quantum dots (QDs) have unique optoelectronic properties^24^,^25^, such as the colour tunability^26^,^27^,^28^,^29^, narrow emission spectra^30^, high quantum yield^31^ and photo/air stability^32^. Additional advantages include printability on various substrates^33^,^34^, ultra-thin active layers^35^ and high luminescence at low operating voltages in QLEDs^36^,^37^,^38^. However, previously reported QLEDs are not suitable for wearable displays because they are not deformable in multiple directions. In addition, for full-colour wearable QLED displays, the red–green–blue (RGB) subpixels should be precisely aligned with high resolution, which cannot be realized by the conventional solution processes^39^,^40^. Dry transfer printing provides an effective route to fabricate pixelated RGB QD films over a large area, but the previous printing methods^41^,^42^,^43^,^44^ using structured stamps have severe discrepancies between the original designs and the resulting pixel shapes, particularly in high-definition designs.

Here, we report ultra-thin and wearable RGB QLED arrays based on the high-resolution intaglio transfer printing technique. This novel transfer printing process utilizes an intaglio trench to create full-colour QD arrays with controlled and uniform pixel sizes that can achieve 2,460 pixels per inch (p.p.i.) resolution. These aligned RGB pixels can be employed to manufacture efficient true-white LEDs or active matrix-driven full-colour displays. Furthermore, high-efficiency deformable QLEDs (brightness of 14,000 cd m^−2^ under the low driving voltage of 7 V, which is the best device performance among the wearable LEDs reported so far^6^,^10^,^14^,^15^) are applied in electronic tattoo demonstrations as a practical example of wearable devices. Ultra-thin designs enable QLEDs to conform to various curvilinear and dynamic surfaces and maintain high EL performances after 1,000 repeated deformation tests.

## 

### High-resolution intaglio transfer printing

For high-definition full-colour RGB QLED arrays, a novel QD integration process, known as the intaglio transfer printing, has been developed through which nanocrystal (NC) layers can be transfer-printed on various substrates regardless of the size, shape and arrangement of pixels. The procedure is illustrated in Fig. 1a. The QD layer coated on the donor substrate was quickly picked-up with a flat elastomeric polydimethylsiloxane (PDMS) stamp (Fig. 1a, process (1)). The picked-up QD layer was lightly contacted on the intaglio trench (Fig. 1a, process (2)) with a pressure of <50 g cm^−2^ and slowly detached<1 mm s^−1^ (Fig. 1a, process (3)). Only the non-contacted part of QD layer remained on the stamp and was transfer-printed on the target substrate (Fig. 1a, process (4)). This transfer printing is facilitated by the differences in surface energy between PDMS stamp and the target substrates (19.8 mJ m^−2^ for the PDMS and >200 mJ m^−2^ for the glass, organic layers and oxide layers) on which the QD layer can be tightly bound. On the basis of the same principle, multiple transfer printings are also possible (Fig. 1a, processes (5) and (6)); the second QD layer is exquisitely integrated on the first layer without any morphological changes. The resulting photoluminescence (PL) image is shown in Fig. 1b. The optical microscope images (Fig. 1c) and fluorescence microscope images (insets) show magnified views of each colour pattern in Fig. 1b, which consists of tens of micron-sized pixels (triangle, hexagon and star patterns). High-resolution aligned RGB pixels, ranging from 441 p.p.i. (30 μm pixel size) to 2,460 p.p.i. (6 μm pixel size; magnified view in inset), can be created by the multiple printing processes described above (Fig. 1d), demonstrating that the novel method is applicable to ultra-high resolution full-colour QD displays.

As the pixel size decreases, the intaglio transfer printing technique becomes more important. We compare the results obtained from the intaglio transfer printing (current) and structured stamping (conventional) methods (Fig. 2a–e). See Fig. 1a (intaglio printing) and Supplementary Fig. 1 (structured stamping) for comparison of the processes. The red boxes and white areas represent the designed patterns and transferred QDs, respectively (Fig. 2a). The fraction of the non-transferred area in the structured stamping method increases at higher resolution (Fig. 2a,b; representative images and statistical data). On the contrary, the intaglio transfer printing process accomplishes the transfer yield of ∼100% (see more transfer printing results of array configurations with various resolutions in Supplementary Fig. 2). The same tendency is observed in different shapes (circular dots and spaced lines; Supplementary Figs 3 and 4), demonstrating ∼100% transfer yield regardless of the size or shape of the patterns. The discrepancies from the designed patterns are particularly dominant near the edges of dot (square and circle) patterns, rather than line-and-space patterns (Fig. 2b,c, Supplementary Figs 2–4). The importances of fine dot patterns are particularly highlighted in patterning complex RGB pixels in full-colour displays.

Theoretical analysis of the enhanced yields of high-resolution patterning in the intaglio transfer printing over the structured stamping was performed using the finite-element method (FEM). Supplementary Fig. 5a–d compares two methods by simulating the transfer printing of a square pixel (size: 150 × 150 μm). In the structured stamping method, the shape is determined by the pick-up process (process (1) and (2) of Supplementary Fig. 1). As the contacted structure stamp is rapidly retrieved, the delamination between the stamp and the QD layer is initiated from edges of the stamp structure and propagates into the centre of the stamp structure, which induces stresses and generates cracks in the QD layer (Supplementary Fig. 5e). Cracks of the QD layer, therefore, occur at the inside of designed pixel edges and result in a reduced pixel size (Fig. 2d). On the contrary, in the intaglio transfer printing method, the pixel shape is determined by the QD release process from the flat stamp to the intaglio trenches (process (2) and (3) of Fig. 1a). Cracks of the QD layer occur at sharp edges of intaglio trenches (Supplementary Fig. 5f). Therefore, the obtained pixel pattern precisely matches the original design (Fig. 2e). The QD/intaglio trench interfacial energy, which is much higher than the QD/stamp interfacial energy, further helps the high definition and yield. See Supplementary Methods for details of FEM simulations and related mechanical analysis.

The intaglio printing process can be generalized to transfer various QD layers (Supplementary Fig. 6) regardless of QD materials (CuInSe and PbS) or sizes (2–18 nm). Furthermore, the current method is readily expanded over large areas by the repetitive aligned transfer printing, which is a critical technology for the mass production (Fig. 2f). Often, distances between pixels should be variable depending on pattern designs. The structured stamping method shows the sagging and leaning of structures in elastomeric stamps, thereby showing low yields, particularly with a large pattern spacing (Supplementary Fig. 7a,c). However, the intaglio stamping method does not exhibit these defects (Supplementary Fig. 7b,c).

### White LEDs fabricated by transfer printing of RGB QDs

Our intaglio transfer printing technique can be utilized to create high-performance pixelated white QLEDs (PWQLEDs) on flexible substrates (Fig. 3a). Conventional white QLEDs have employed a mixture of several kinds of QDs and phosphors of different characteristic wavelengths^45^,^46^,^47^,^48^,^49^. However, these white QLEDs have been proven to be inefficient owing to the inevitable energy transfer between the different QDs/phosphors (for example, Förster energy transfer)^50^,^51^. In the mixed system, it is difficult to obtain balanced white light because the energy transfer occurs from B to G, R and from G to R. Therefore, it is desired to realize white emission by controlling the injected current of each RGB subpixel in the pixelated LED arrays, rather than by controlling RGB luminophore content in the mixed system.

On the other hand, the current flexible PWQLEDs utilize aligned RGB fine pixels (Fig. 3b), whose colour can be tuned to be the true white with high efficiency. We unify QD materials using CdSe/ZnS alloyed QDs (Supplementary Fig. 8) to minimize variations in the RGB EL brightness and to prevent the inefficient blue EL of CdS-based QDs^35^,^42^. All the CdSe/ZnS alloyed QDs have the same type of ligand, oleic acid (Supplementary Fig. 9). Figure 3c shows the band diagram for PWQLEDs, which is estimated from the ultraviolet photoelectron spectra (Supplementary Fig. 10). Band alignments and efficient electron and hole injections are enabled by the careful selection and integration of inorganic/organic materials for each layer.

The EL of PWQLEDs consists of three distinct peaks that match each monochromatic RGB EL (Fig. 3d). The EL location of PWQLEDs in Commission International de l'Éclairage coordinates is (0.39, 0.38) under 6 V bias, which indicates the emission of true-white light (Fig. 3e). The EL spectra at different applied voltages are presented on Supplementary Fig. 11. Furthermore, EL efficiencies are compared between PWQLEDs and mixed white QLEDs (MWQLEDs) in which the active layer is created by mixing RGB QDs in the solution phase (Supplementary Methods for fabrication details). The brightness of PWQLEDs is enhanced over MWQLEDs by ∼10 to ∼52% depending on the applied voltage (Fig. 3f), and the EQE of PWQLEDs is higher than that of MWQLEDs in entire operating voltage (Fig. 3g). In addition, flexible PWQLEDs present stable current density versus voltage (*J–V*) characteristics under various bending angles (Fig. 3h).

For the better understanding of the enhanced performance of PWQLEDs, time-resolved PL measurements were conducted for QD layers employed in MWQLEDs and PWQLEDs (Fig. 3i–k; data at the blue, green and red wavelengths and a summarized plot, respectively). The time-resolved PL of each RGB QD layer was also measured for the comparison. In MWQLEDs, the carrier lifetime of blue and green QDs significantly decreases, while that of red QDs increases, which implies the energy transfer between QDs^50^,^51^. Because QDs with different band gaps are adjacent to each other in the close-packed (mixed) layer, they transfer energy to neighbouring QDs with lower energy band gaps instead of emitting photons. The energy transfer between QDs of the same colour is neglected for analysis. In PWQLEDs, on the contrary, the carrier lifetime of pixelated QD arrays does not change from that of individual RGB QDs. These results demonstrate that the geometrical separation of pixelated configurations effectively suppresses the energy transfer process, enabling highly efficient true-white emission.

### Electronic tattoos based on ultra-thin and wearable QLEDs

The current QLED technologies are applied in electronic tattoo demonstrations (Fig. 4). Ultra-thin form factors (total thickness of ∼2.6 μm, including ∼300-nm-thick active and ∼1.1-μm-thick encapsulation layers; inset of Fig. 4a) enable various deformations and conformal integrations with soft, curvilinear epidermal tissues^2^,^7^. The detailed device structures and the magnified view of active layers (electron transport layer (ETL), QDs and hole transport layer) are shown in Fig. 4a,b, respectively. The ultra-thin encapsulation consists of a Parylene-C and epoxy bilayer. Electronic tattoos show outstanding device performances, such as a high brightness of 14,000 cd m^−2^ at a driving voltage of 7 V and EQE of 2.35% at 4.5 V bias (*J–V–L* characteristics, Fig. 4c). The electronic tattoo exhibits EQE above 1% in the range of 3.6–6.9 V applied voltages (current density: 3.4–1,132 mA cm^−2^) as shown in Supplementary Fig. 12a. To the best of our knowledge, the brightness is higher than the previously reported values of the wearable LEDs at the same driving voltage^6^,^10^,^14^,^15^. The high device performance at the low driving voltage, which can be obtained by commercial mobile batteries, is particularly beneficial to wearable device applications. The high EL performance remains stable after 1,000 cycles of uniaxial stretching (∼20% applied strain, Fig. 4d). For stretching tests, ∼20% prestrain, which is similar with the skin stretchability^7^, is applied to ultra-thin QLEDs to form a wavy structure^9^. Moreover, as shown in Supplementary Fig. 12b, the lifetime of electronic tattoo is about 41.7 h at 3 mA applied current (initial brightness=4,554 cd m^−2^), which corresponds to device lifetime of 12,815 h at 100 cd m^−2^ (lifetime × initial brightness^1.5^=constant)^20^. Furthermore, these ultra-thin QLEDs can be laminated on various curvilinear substrates, such as the crumpled Al foil, human skin, round glass, metal can and sharp edges of a slide glass (Fig. 4e–g, Supplementary Fig. 13a–d). Various deformations, such as bending, folding or crumpling, as well as moistures (water droplets) do not cause mechanical/electrical damages or any decrease in the EL performance (Fig. 4e–g, Supplementary Fig. 13e, Supplementary Movie 1). The current electronic tattoo platform can be extended to wearable PWQLEDs that are laminated on the human skin (Fig. 4h).

## Discussion

In conclusion, we demonstrate ultra-thin, wearable RGB LED arrays fabricated using colloidal QDs and high-resolution intaglio transfer printing technology. This novel pixel-defining technique achieves the 60 K ultra-high-definition RGB resolution (based on 40-inch flat panels). High-efficiency true-white QLED arrays as well as electronic tattoo applications demonstrate versatile utilities of the current work. These state-of-art devices can be laminated on various soft and curvilinear surfaces without diminishing the high EL efficiency. The current progress will realize high-definition full-colour deformable QLEDs and enable design variations in emerging wearable electronics.

## Methods

### Materials

The CdSe/ZnS QDs for blue and green QDs and the CdSe/CdS/ZnS QDs for red QDs were synthesized in the laboratory. All QDs have CdSe–ZnS core-shell alloyed structures to enhance EL and show a PL quantum yield of >∼80%. The synthesis methods for the colloidal NCs are described in Supplementary Methods. Poly(3,4-ethylenedioxythiophene): poly(styrenesulfonate) (PEDOT:PSS; VP AI 4083) was purchased from Clevios, and TFB (poly[(9,9-dioctylfluorenyl-2,7-diyl)-co-(4,4′-(*N*-(4-sec-butylphenyl))diphenylamine)], SOL 2036) was purchased from Solaris. Anhydrous butanol, heptane and m-xylene were purchased from Sigma-Aldrich. Zinc oxide NCs for the ETL, PbS QDs and CuInSe QDs were synthesized in the laboratory (details in Supplementary Methods). Transmission electron microscopy images were obtained on a JEOL 2100F electron microscope. The absorption spectra were acquired on a CARY 5000E ultraviolet–visible–near-infrared spectrophotometer. PL and time-resolved fluorescence spectra were recorded on an FLS 980 spectrometer (Edinburgh Instruments). For PL, the QDs were excited with a steady-state xenon lamp, and the emitted photons were detected by a single-photon-counting photomultiplier. The valance band maximum of the layer materials was determined by the ultraviolet photoelectron spectroscopy (Thermo Fisher Scientific Co.) with a He discharge lamp (21.2 eV).

### Intaglio transfer printing

The QD layer was spin-casted on the 1-octadecyltrimethosysilane-treated silicon (Si) substrate (donor substrate), and a flat PDMS stamp was utilized to quickly pick up (10 cm s^−1^) the QD layer^41^. The picked-up QD layer was conformally contacted on the intaglio trench with the low applied pressure and slowly detached. Then the intaglio QD patterns are formed on the stamp. Finally, the non-contacted QD layer, that is, intaglio patterns, remaining on the stamp can be transferred to the desired substrate. The intaglio trenches were negatively carved on the Si substrate using the deep Si etcher (Versaline, PLASMA THERM). The residual QD layers on the trench were easily eliminated through either the mechanical rubbing or cleaning in piranha solution; thus, intaglio trenches are reusable.

### Fabrication of PWQLEDs

To fabricate PWQLEDs, ∼100-nm-thick indium tin oxide (ITO) on polyethylene terephthalate substrate was patterned and successively cleaned with the acetone, isopropyl alcohol, deionized water and isopropyl alcohol. After the 5 min of ultraviolet ozone (UV/O_3_) treatment, an anode substrate was spin-coated with Poly(3,4-ethylenedioxythiophene): poly(styrenesulfonate) (PEDOT:PSS) followed by annealing at 120 °C for 10 min. Then, TFB was spin-coated at 2,000 r.p.m. and annealed at 150 °C for 30 min. Each RGB QD pixel was precisely patterned on the annealed TFB layer by the intaglio transfer printing. A custom-made transfer printer, which could control the pressure and pick-up rate, was used to pick up QD layers from the donor substrate. A manual-type mask aligner was used for the delicate alignment of RGB pixels on the TFB layer. The area ratio of RGB pixels in PWQLEDs was 1:1:2. After transferring the QD layer, the device was annealed at 150 °C for 30 min. A 40-nm-thick ZnO NC layer was utilized for the ETL, which was spin-coated on the transferred QD layer, followed by annealing at 120 °C for 10 min. A 50-nm-thick lithium–aluminium alloy cathode was then thermally evaporated at a rate of 0.1 nm s^−1^ through a shadow mask. Final encapsulations are added for the protection.

### Fabrication of wearable QLEDs

The nickel sacrificial layer was evaporated on a cleaned Si wafer. The bottom encapsulation film was composed of double-layered Parylene-C and epoxy (SU8-2000.5, MicroChem), the spin-coated 600-nm-thick epoxy on the Ni-coated Si wafer followed by evaporated 500-nm-thick Parylene-C. The film was then annealed at 95 °C for 1 min and then at 150 °C for 30 min after ultraviolet exposure. The Parylene-C layer protects QLEDs from the oxidation, and the epoxy layer prevents the Parylene-C film from the delamination and provides an ultra-flat surface through the reflowing process. ITO was sputtered on the encapsulation film (50 W, 30 min, 5 mTorr, 200 °C) and patterned. Then, PEDOT:PSS was spin-casted on the ultraviolet/O_3_-treated cathode at 2,000 r.p.m. for 30 s. The resulting cathode was annealed at 120 °C for 10 min in the ambient atmosphere and annealed at 150 °C for 10 min in a glove box to remove the residual solvent. TFB (0.5 wt%) in m-xylene was spin-coated and annealed at 150 °C in the glove box. The QD layer was transfer-printed on the ITO pattern by the intaglio transfer printing method in air and annealed at 150 °C in the glove box. In addition, Zinc oxide (ZnO) NCs in butanol were spin-coated and annealed at 145 °C. Finally, the anode material, Li/Al alloy, was thermally evaporated for ∼80 nm and encapsulation layers (Parylene-C and epoxy layers) were deposited to protect the device. The encapsulated device was flooded in the Ni etchant to dissolve the sacrificial layer. For the buckled, stretchable QLEDs, ultra-thin QLEDs were transfer-printed on the prestretched PDMS layer, followed by a mild annealing treatment to dry any residual water. Current flows through the vertically overlapped area between ITO and Li/Al.

### PL lifetime measurements

Time-resolved PL spectroscopy data were obtained by a time-correlated single-photon-counting module in the FLS 980 spectrometer (Edinburgh Instruments, UK). The individual R/G/B QDs, RGB mixed QDs and RGB aligned pixelated QDs on quartz substrates were excited using a ∼376-nm laser (EPL-375). The pulse width and repetition rate of the pulsed diode laser were ∼74.5 ps and ∼5 MHz, respectively. The PL was spectrally dispersed in a monochromator (1,800 g mm^−1^ grating) and detected with a microchannel plate photomultiplier (detector response width <25 ps) at the emission maxima of each colour (440, 520 and 640 nm for blue, green and red, respectively). The pulsed laser and time-correlated single-photon-counting system provided a time window of 50 ns with 8,192 data channels.

### Device characterization

The current–voltage curves for QLEDs were measured with a Keithley 2436 source metre. The EL of QLEDs was measured with a CS-2000A spectrophotometer (measurement spot size <500 μm) by sweeping the applied voltage from 0 to 8 V. The white QLED and flexible QLED measurements were performed at room temperature in the glove box, and all stretchable and deformable QLED measurements were performed in ambient conditions. All the device characteristics were measured and averaged with six different devices and 12 different LED units.

## Additional information

**How to cite this article:** Choi, M. K. *et al*. Wearable red–green–blue quantum dot light-emitting diode array using high-resolution intaglio transfer printing. *Nat. Commun.* 6:7149 doi: 10.1038/ncomms8149 (2015).

## Supplementary Material

Supplementary Figures, Supplementary Methods and Supplementary References Supplementary Figures 1-13, Supplementary Methods and Supplementary References

Supplementary Movie 1 Stretchable electronic tattoo.

## Figures and Tables

**Figure 1:**
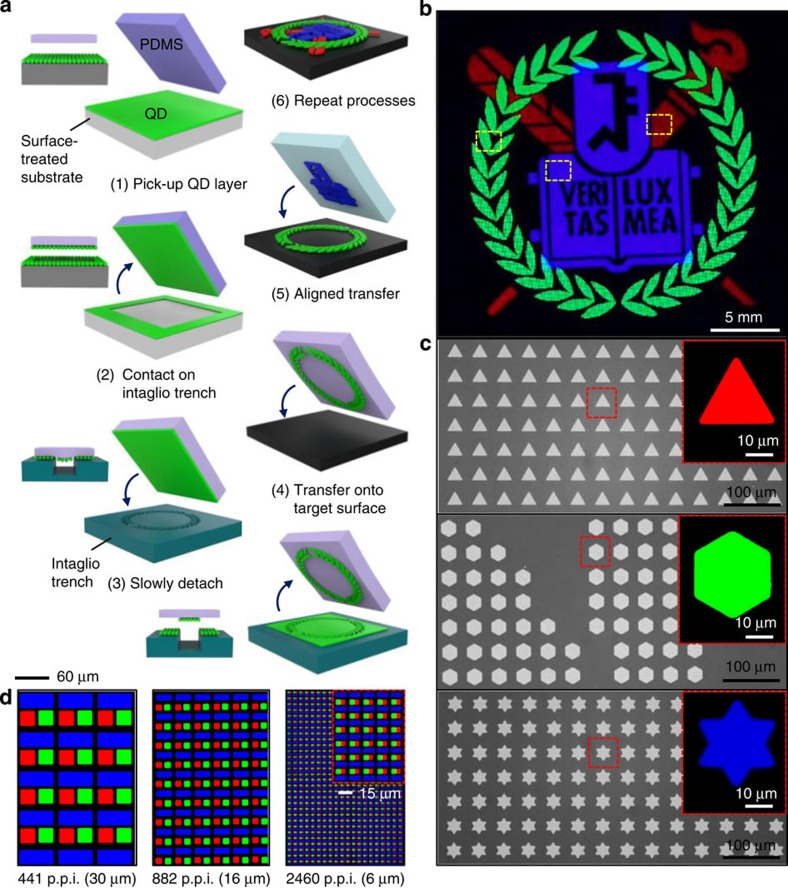
Intaglio transfer printing for high-resolution RGB QLEDs. (**a**) Schematic illustration of the intaglio transfer printing process. Inset images on the left of each frame show the side view. (**b**) The PL image of the RGB QD patterns via multiple aligned transfer printings. (**c**) Magnified views of selected regions of **b**. Each colour pattern consists of thousands of tens-of-microns-sized pixels (red: triangle (top), green: hexagon (middle) and blue: star (bottom)). Insets show further magnified PL images of pixels. (**d**) The PL images showing aligned RGB pixels whose resolution is between 441 p.p.i. (left) and 2,460 p.p.i. (right).

**Figure 2:**
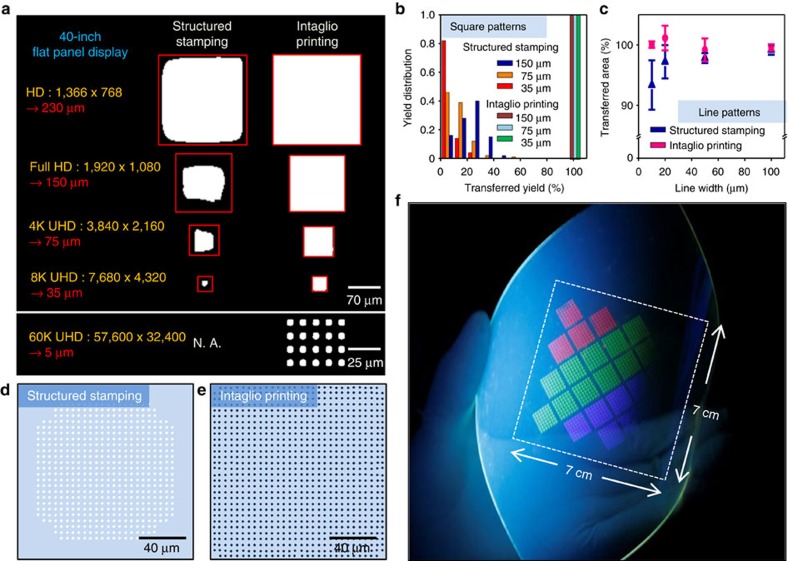
Experimental and theoretical analysis of the intaglio transfer printing. (**a**) Pattern size scaling in the structured stamping (left) and intaglio transfer printing (right). QD transfer yields of the structured stamping dramatically decrease especially in high resolutions, while those of the intaglio printing approach ∼100% in all design rules. (**b**) Distribution of transfer printing yields at different pattern sizes (150, 75 and 45 μm). The transfer printing yield for the structured stamping dramatically decreases with the pattern size, while that of intaglio printing maintains ∼100%. Detailed results are shown in Supplementary Fig. 2. (**c**) Percentile proportion of the transferred QD line pattern area to the original pattern area. As the line width decreases from 100 to 10 μm, the structured stamping yield decreases, while intaglio printing maintains ∼100%. Detailed results are shown in Supplementary Fig. 4. (**d**,**e**) FEM simulations of the transferred area of the rectangular pattern (size: 150 × 150 μm) for the structured stamping (**d**) and intaglio printing (**e**). (**f**) PL image of a large-area QD dot array (7 × 7 cm) patterned by repeated aligned intaglio transfer printing on a flexible polyethylene terephthalate substrate.

**Figure 3:**
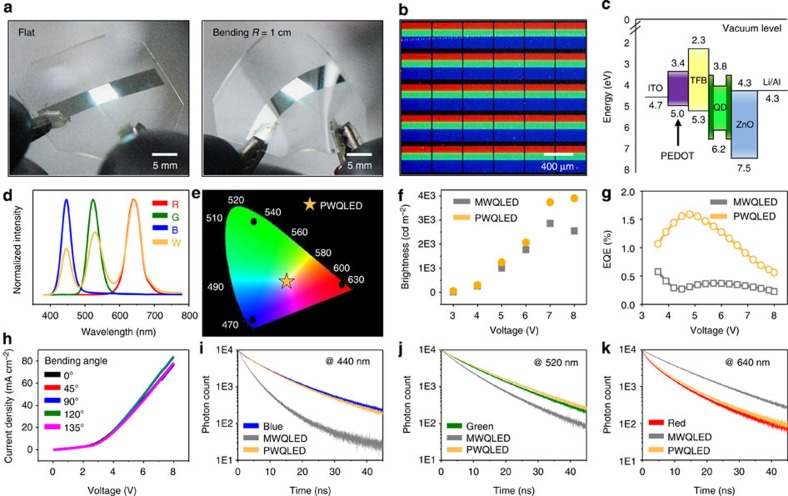
True-white light emission based on pixelated RGB QLEDs. (**a**) Optical images of the flexible white QLEDs under the bias. (**b**) Magnified view (PL image) of the RGB QD pixels of white QLEDs. (**c**) Energy band diagram of white QLEDs estimated by the ultraviolet photoelectron spectrometry. (**d**) EL spectra of PWQLEDs and each monochromatic (R, G and B) QLED. (**e**) CIE 1931 *x*–*y* chromaticity diagram showing the true-white colour (0.39, 0.38) of PWQLEDs. (**f**) Brightness versus voltage of PWQLEDs and MWQLEDs. PWQLEDs show the higher efficiency than MWQLEDs, particularly at the high brightness. (**g**) External quantum efficiency of PWQLEDs and MWQLEDs. (**h**) Electrical properties (*J–V* characteristics) at different bending angles. (**i**–**k**) Time-resolved PL spectra of aligned RGB (PWQLED), mixed (MWQLED), and monochromatic (R, G and B) QD layers.

**Figure 4:**
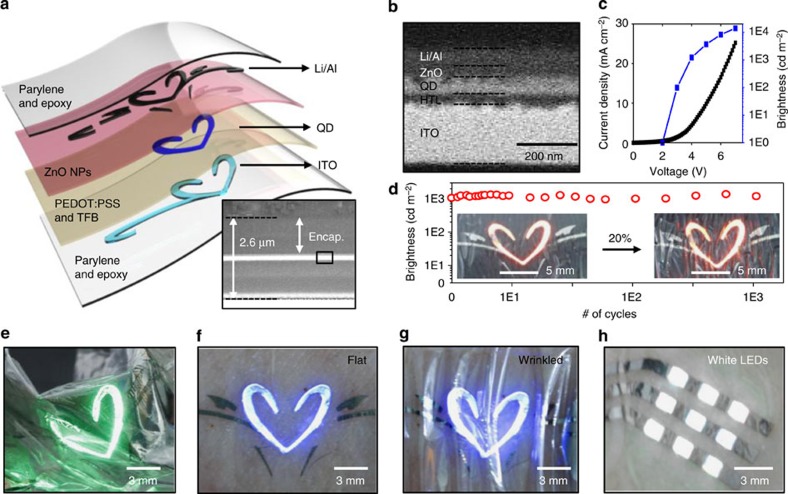
Electronic tattoo demonstrations using ultra-thin wearable QLEDs. (**a**) Exploded view of the electronic tattoo, which shows the layer information of the device. The inset is a cross-sectional scanning electron microscope (SEM) image of the electronic tattoo in which the thickness of the encapsulation and active layers are shown. (**b**) Magnified view of the cross-sectional SEM image (inset of Fig. 4a) that shows the detailed layer information of active layers. (**c**) The *J–V–L* characteristics of the ultra-thin, wearable QLEDs. (**d**) Stable brightness in multiple stretching experiments (∼20%, 1,000 times). The inset shows photographs of buckled and stretched ultra-thin red QLEDs (0 and ∼20%, left and right). (**e**) Optical image of ultra-thin green QLEDs laminated on crumpled Al foil. (**f**,**g**) Photographs of the electronic tattoo (blue QLEDs) laminated on the human skin (**f**). The wearable QLEDs maintain the original optoelectronic performances even under skin deformations (**g**). (**h**) Optical image of wearable PWQLED arrays laminated on the human skin.
