# Supplementary material for: Wearable red–green–blue quantum dot light-emitting diode array using high-resolution intaglio transfer printing
Source: Nat Commun. 2015 May 14;6:7149. doi: 10.1038/ncomms8149 (PMC4479007; doi:10.1038/ncomms8149)
Supplement: Supplementary Figures, Supplementary Methods and Supplementary References — Supplementary Figures 1-13, Supplementary Methods and Supplementary References [file ncomms8149-s1.pdf]

Supplementary Information

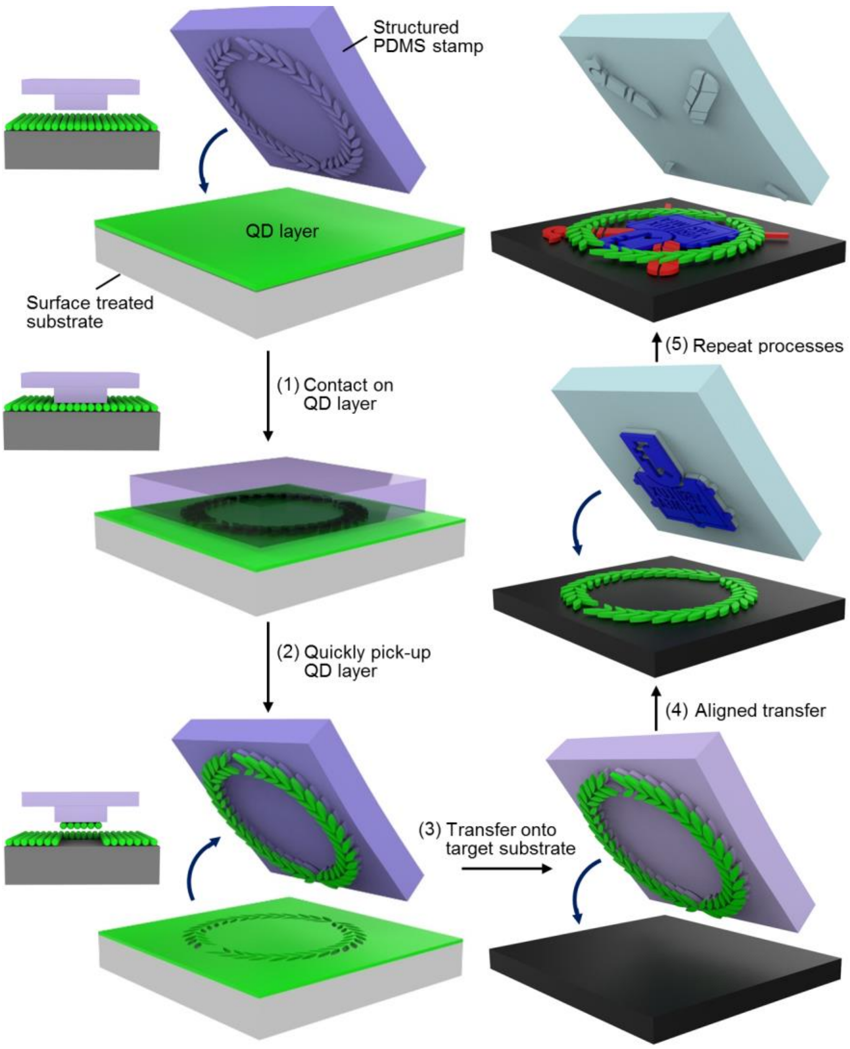

Supplementary Figure 1 | Schematic illustration of the structured stamping method. The QD layer is directly picked up from the ODTS-treated substrate by the structured PDMS stamp. This process is previously reported and widely used as the dry transfer-printing method.

Pixel size (μm)	Structured stamping	Intaglio printing	PPI	40' (TV)
**230**	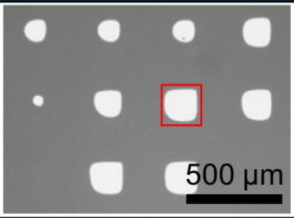	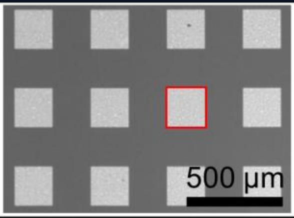	55	-
**150**	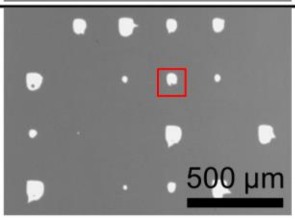	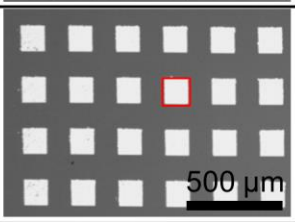	85	FHD
**75**	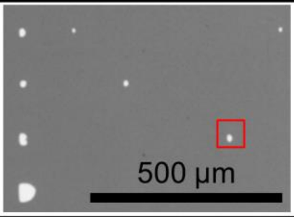	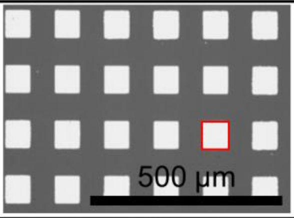	169	4K UHD
**35**	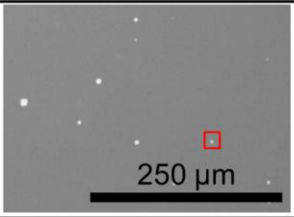	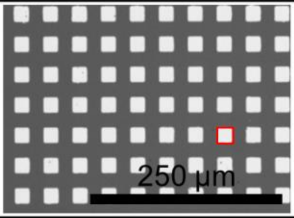	362	8K UHD
**5**	N. A.	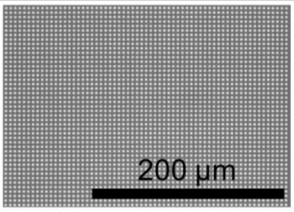	2540	60K UDH

Supplementary Figure 2 | Square pattern-size effect on the yield of transfer printing of QDs. As the designed square pattern size decreases, the transfer yield in the structured stamping method dramatically decreases. On the contrary, the transfer yield in the intaglio printing method approaches ~100% regardless of the pattern size.

Pixel size (µm)	Structured stamping	Intaglio printing	PPI	5' (smart phone)
**100**	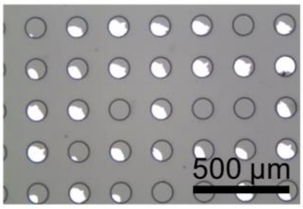	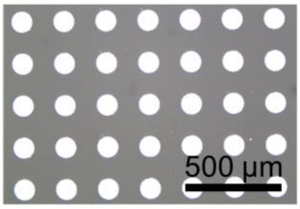	127	-
**45**	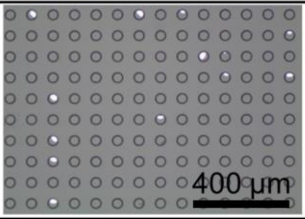	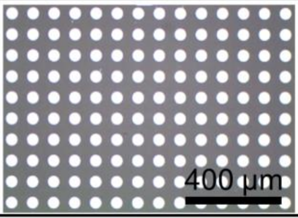	282	-
**30**	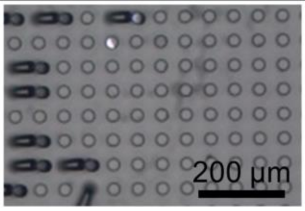	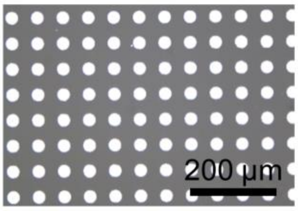	423	Retina
**16**	N. A.	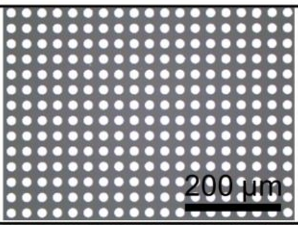	794	FHD
**5**	N. A.	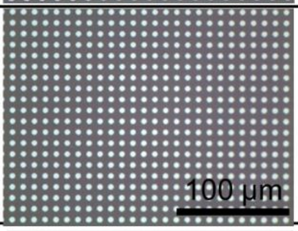	2540	UHD
**<1**	N. A.	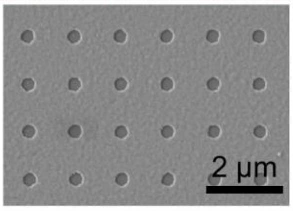	<13,000	µLED

Supplementary Figure 3 | Circular pattern-size effect on the yield of transfer printing QDs. As the designed circular pattern size decreases, the transfer yield in the structured stamping method dramatically decreases. On the contrary, the transfer yield in the intaglio printing method approaches ~100% regardless of the pattern size.

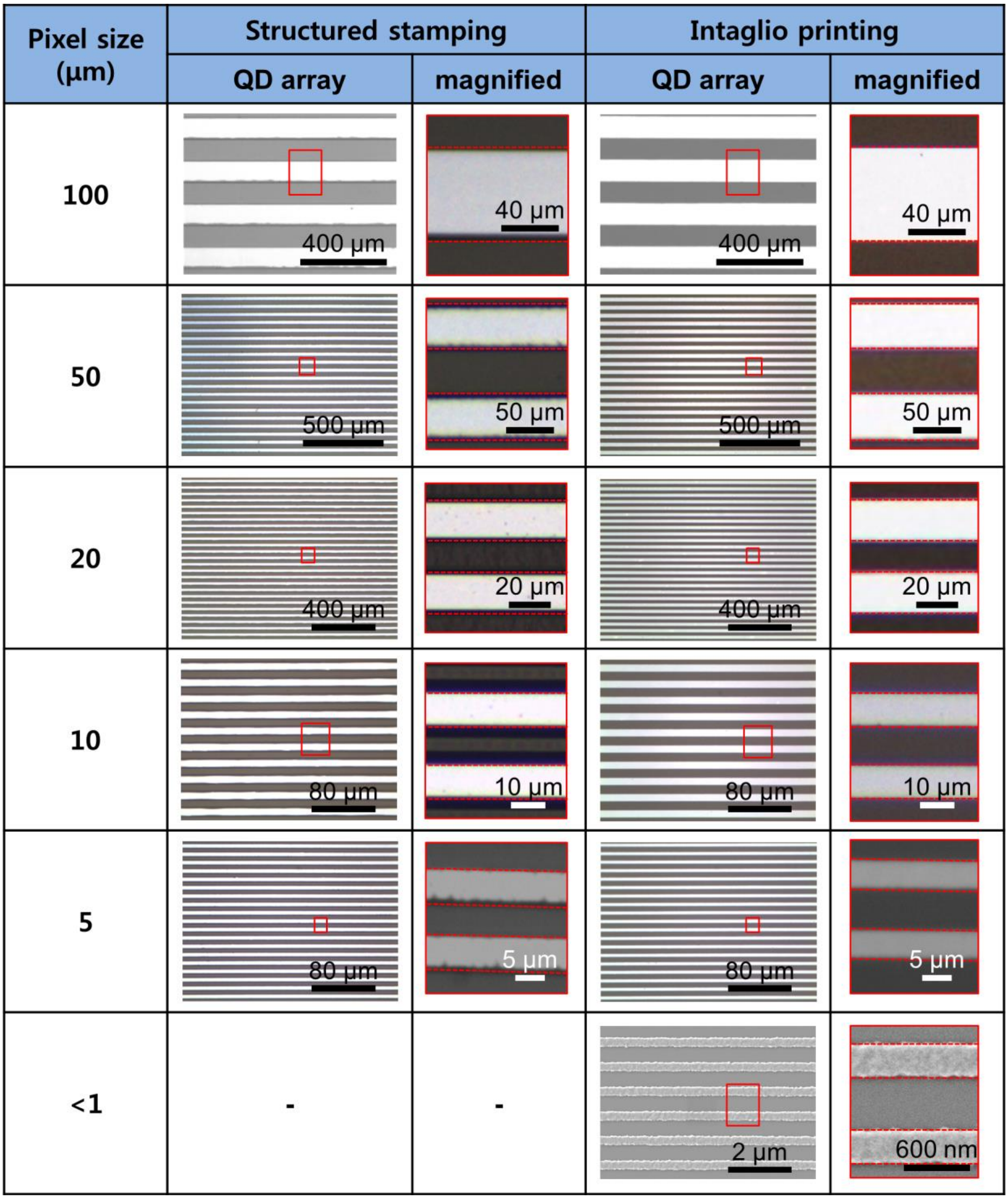

Supplementary Figure 4 | Line-and-space pattern-size effect on the yield of transfer printing QDs. As the designed line-and-space pattern size decreases, the transfer-printing yield in the structured stamping method dramatically decreases. On the contrary, the yield in the intaglio printing method approaches ~100% regardless of the pattern size.

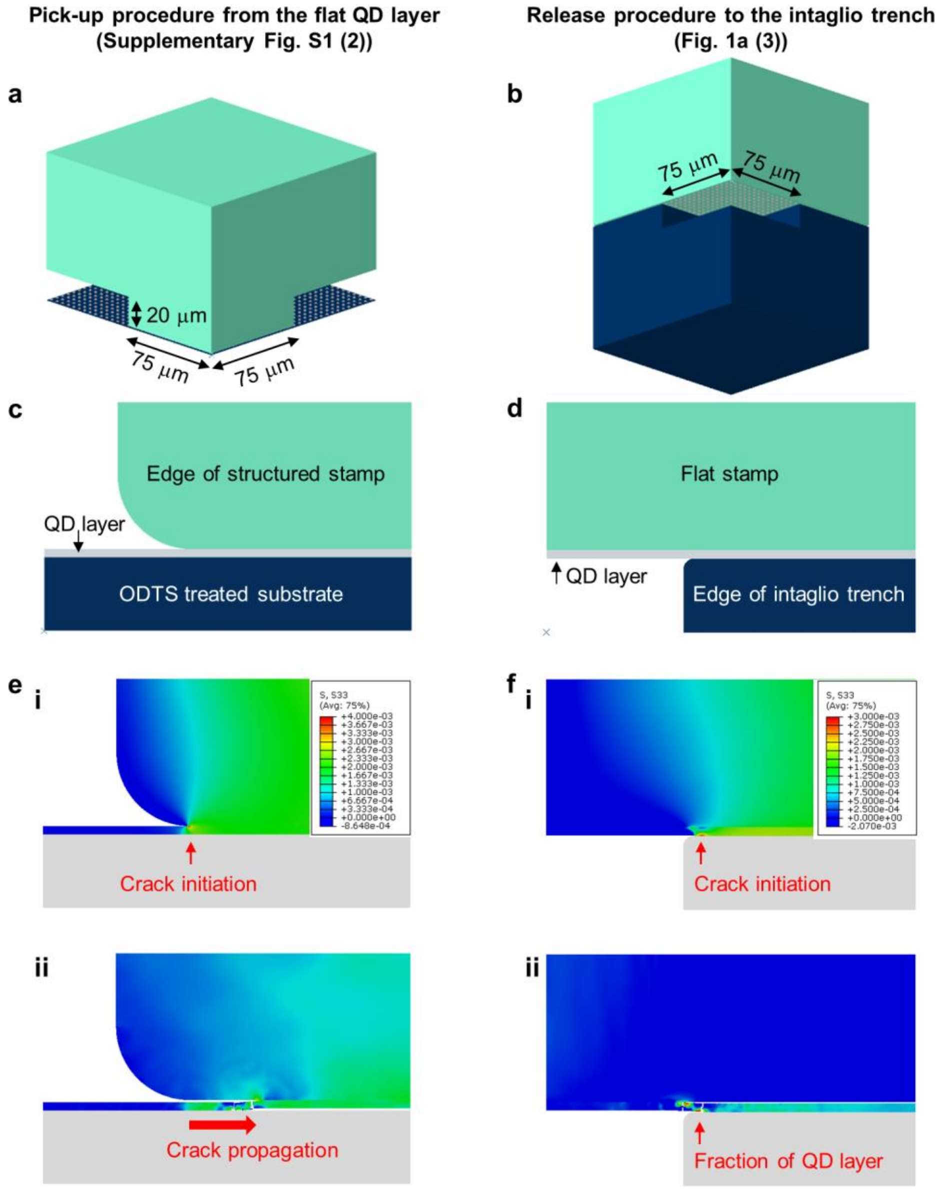

Supplementary Figure 5 | Finite-element method (FEM) simulations of the structured stamping and intaglio printing method. (a, b) One quarter of the single-dot structured stamp **(a)** and the single-dot trench **(b)** contacting the substrate. **(c)** The magnified side-view of the edge of the structured stamp at the pick-up process (process (2) of Supplementary Fig. 1). The structured stamp in contact with the spin-casted QD layer on the ODTS-treated substrate. **(d)** The magnified side-view of the edge of the intaglio trench at the releasing process (process (3) of Fig. 1a). The picked-up QD layer in contact with the intaglio trench. **(e)** Gradient of normal stress on the QD layer and edge of the structured stamp. As the stamp moves upward (pick-up), cracks propagate from the edge into the centre of the stamp (i → ii). **(f)** Gradient of normal stress on the QD layer and the flat elastomeric stamp (i). Cracks do not propagate into the centre, and the QD layer is broken at edge of the trench (ii).

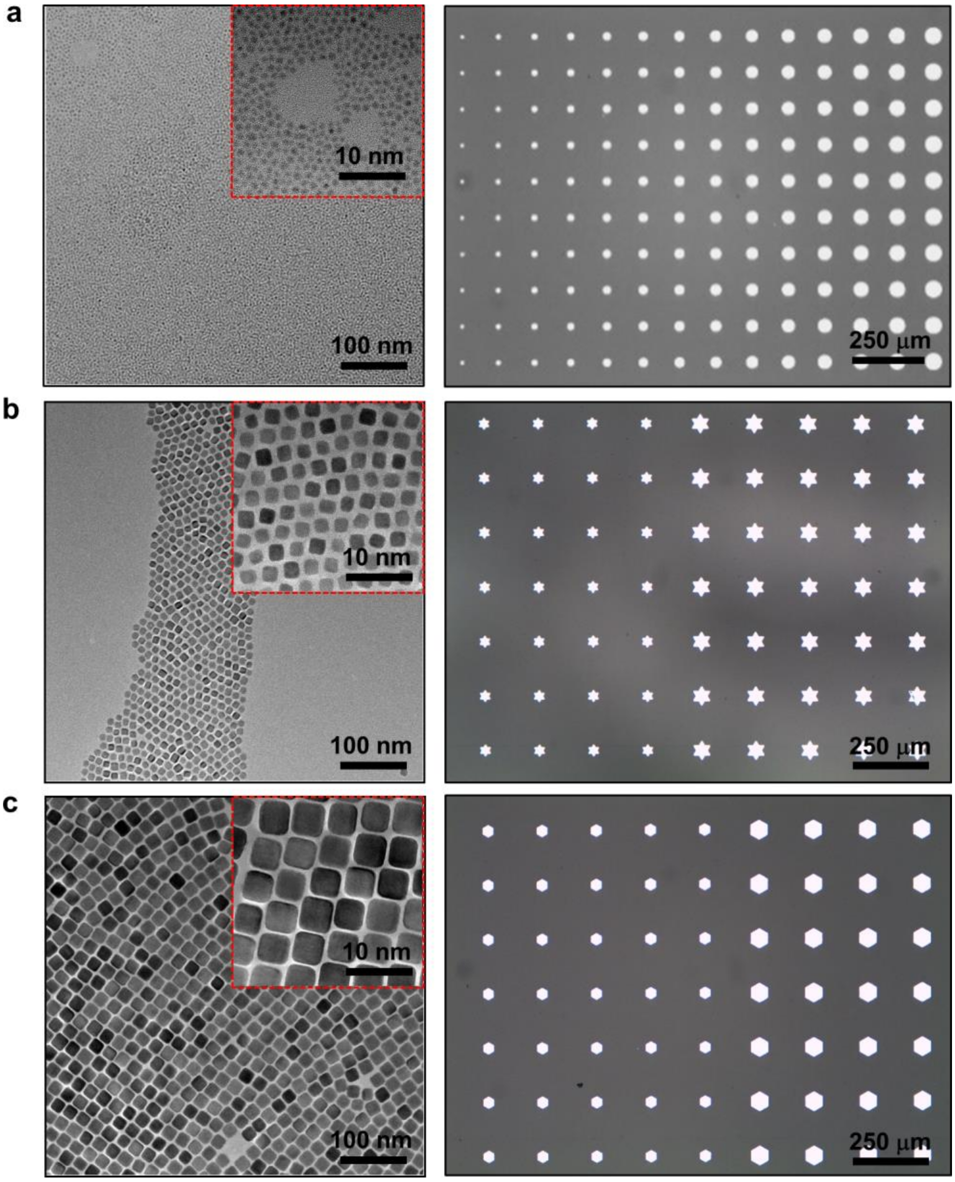

Supplementary Figure 6 | Intaglio transfer printing demonstrations of various QDs. (a) A TEM image of Cu-In-Se QDs (size: 2 nm) (left) and intaglio transfer-printed dot patterns (right) using the Cu-In-Se QD layer. **(b, c)** The TEM images of PbS QDs (8 nm **(b)**, and 18 nm **(c)**) (left) and intaglio transfer-printed patterns (right) using the PbS QD layers. Insets of each image in the left column show the high-resolution TEM images.

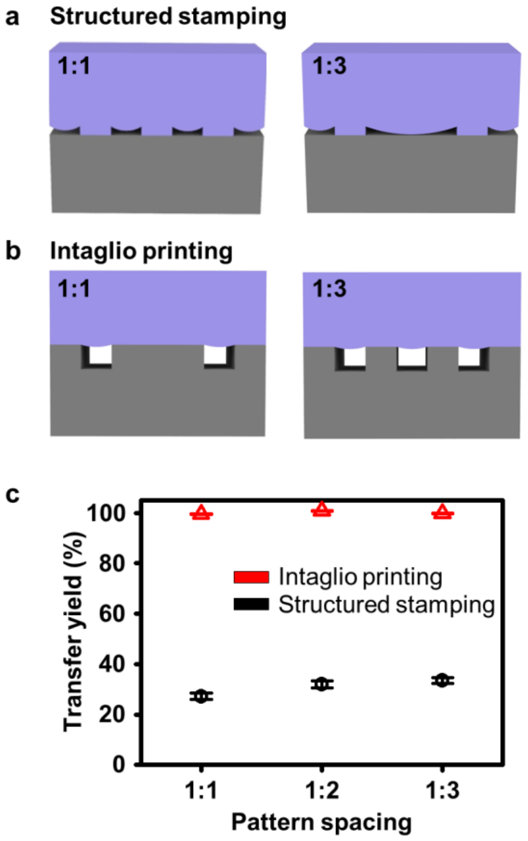

Supplementary Figure 7 | Effect of pattern spacing on the transfer-printing yield. (a) An illustration of the structured stamp with various pattern spacings (pattern:space = 1:1 and 1:3). When the pattern spacing is wide during the pick-up procedure, the stamp may sag or lean because of the high applied pressure, causing deformation in the resulting patterns. **(b)** An illustration of the intaglio trench with various pattern spacings (pattern:space = 1:1 and 1:3). Because the applied pressure on the flat stamp is much lower than that on the structured stamp, deformation of the stamp can be neglected, and the resolution of the pattern is not affected by pattern spacing. **(c)** Transfer yield versus pattern spacing.

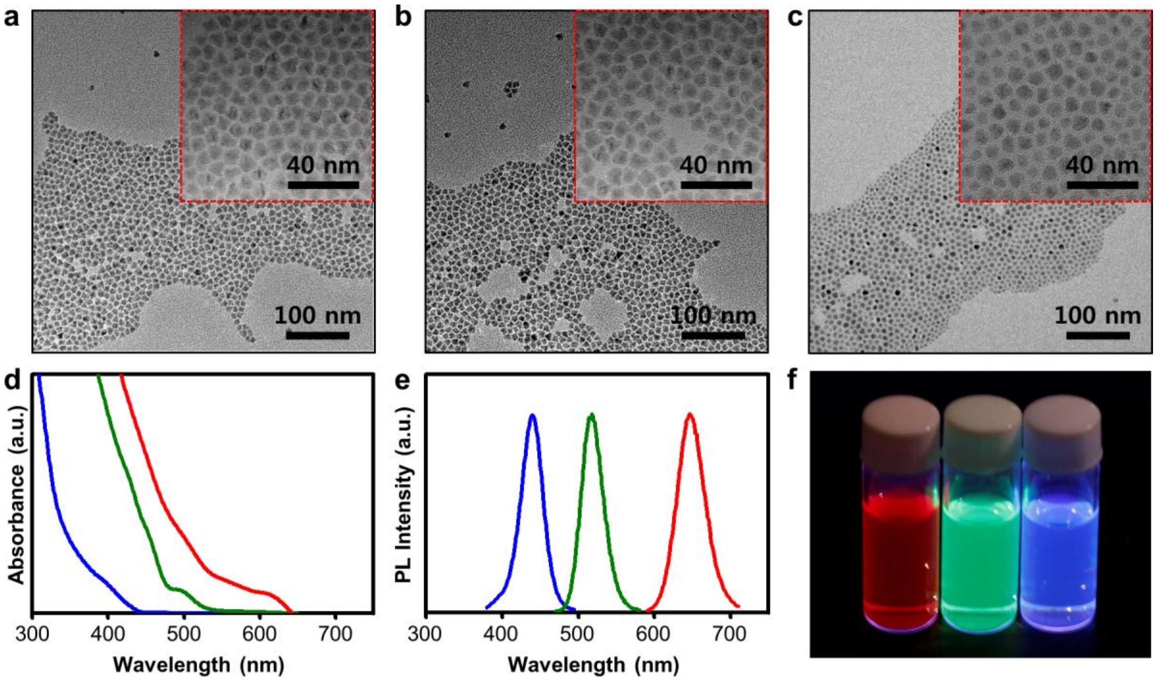

Supplementary Figure 8 | Characteristics of CdSe-based R/G/B QDs. (a–c) The TEM images of the red CdSe/CdS/ZnS QDs **(a)**, green CdSe/ZnS QDs **(b)**, and blue CdSe/ZnS QDs **(c)**. Insets show the high-resolution TEM images. **(d)** Absorption spectra of RGB QDs. **(e)** The EL spectra of RGB QDs. **(f)** Photograph of the RGB QD solution under UV excitation.

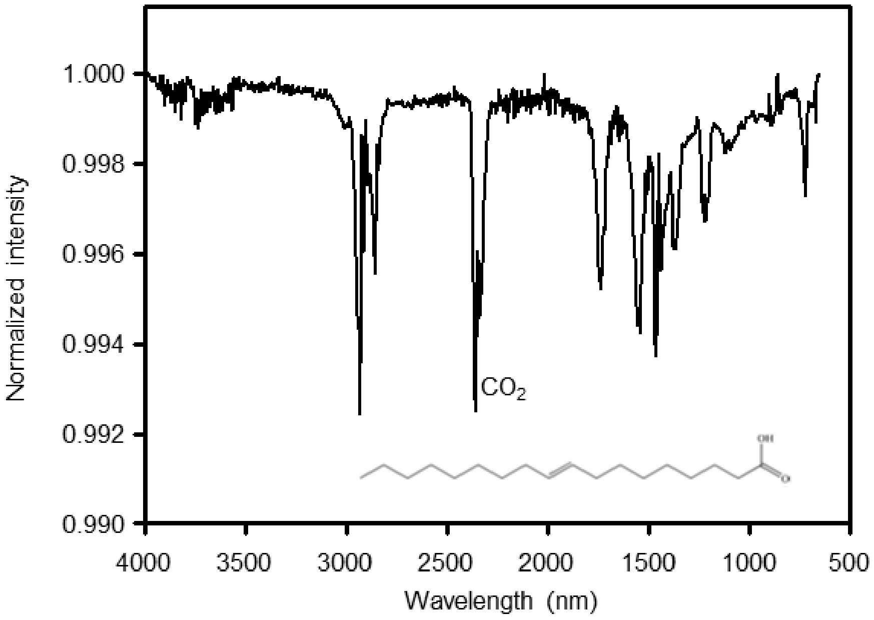

Supplementary Figure 9 | FTIR characterization of CdSe/ZnS alloyed QDs. The FTIR peaks match with the oleate ligands on the surface of the QDs.

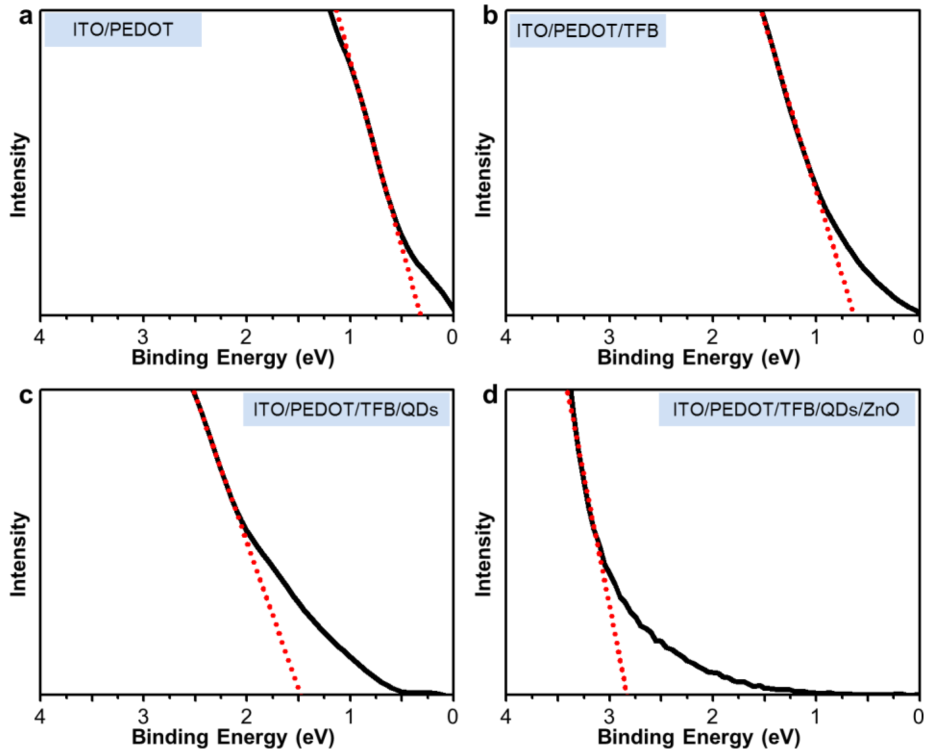

Supplementary Figure 10 | UV photoelectron spectra of layer materials employed in QLEDs. (a–d) The HOMO or valance band maximum of each layer is determined by the edge of the low-binding energy region in UPS spectra: **(a)** ITO/PEDOT, **(b)** ITO/PEDOT/TFB, **(c)** ITO/PEDOT/TFB/QDs, and **(d)** ITO/PEDOT/TFB/QDs/ZnO.

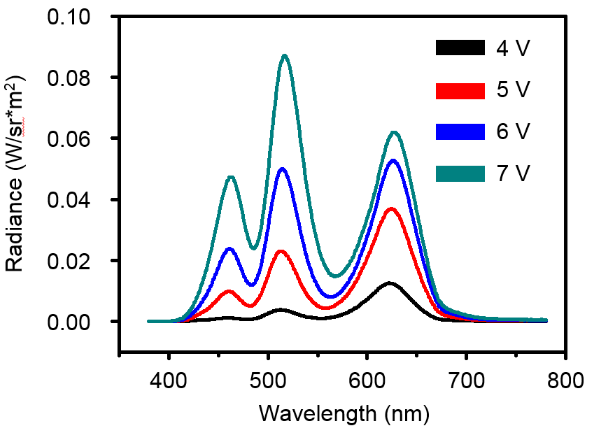

Supplementary Figure 11 | EL spectra of PWQLEDs. As applied voltages change, the relative EL intensities of R/G/B pixels vary.

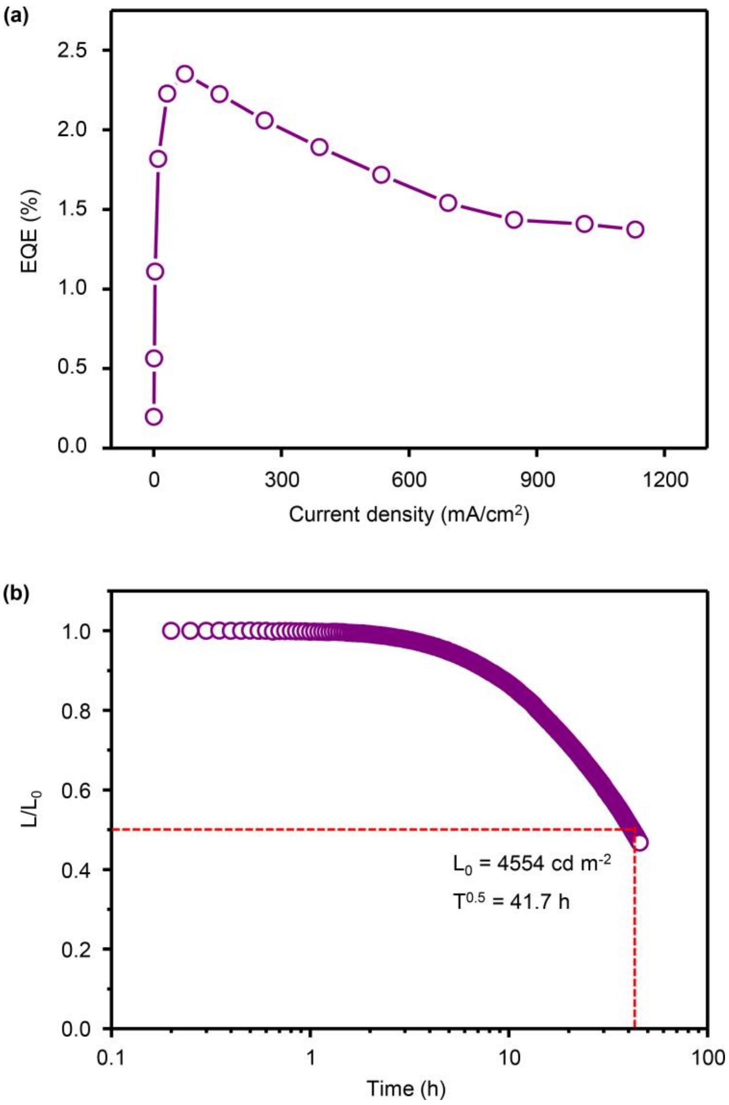

Supplementary Figure 12 | Device characteristics of wearable electronic tattoo. (a) External quantum efficiency (EQE) versus current density. **(b)** Lifetime characteristics. At the initial luminance of 4554 cd m^{-2}, the half-lifetime is 41.7 h under constant current operation (3 mA) at room temperature.

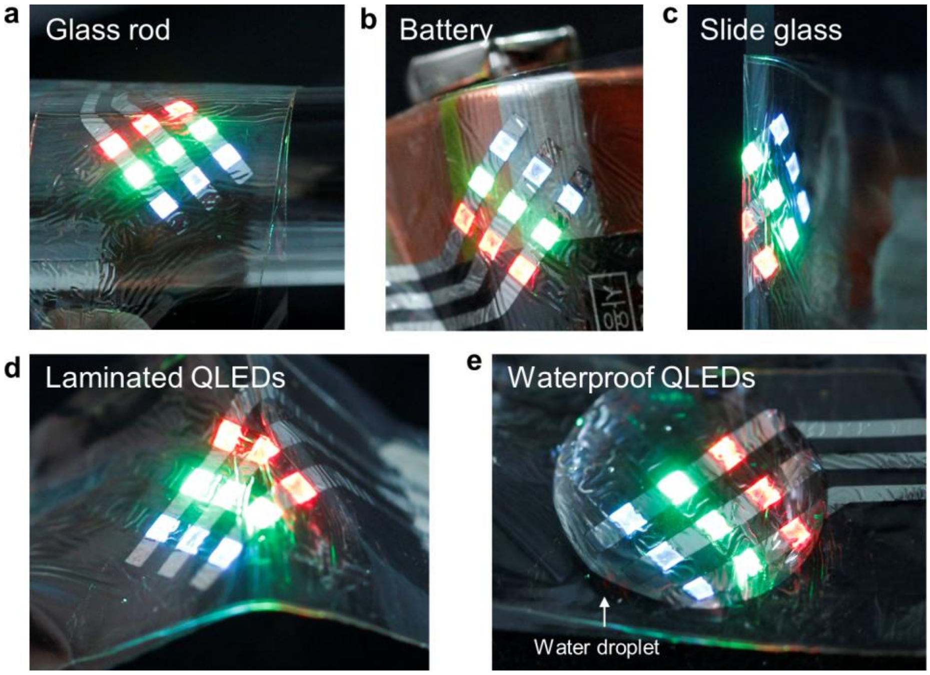

Supplementary Figure 13 | Ultrathin QLEDs on various curvilinear substrates. (a–c) Photographs of the ultrathin and wearable QLED arrays on the curved surface of the glass rod **(a)**, the battery **(b)**, and the edge of the slide glass **(c)**. **(d)** laminated freestanding wearable QLEDs. **(e)** Waterproof wearable QLEDs.

Supplementary Methods

Finite-element method (FEM) simulation of the intaglio transfer printing and structured stamping method

To analyse different yields of the pick-up and transfer printing process, FEM simulations of the structured stamping and of the intaglio transfer printing were used, as shown in Supplementary Fig. 5 as well as Fig. 2d and e. The substrates and stamps were modelled as rigid bodies, and the quantum dot (QD) layer was modelled as a single layer of continuum particle elements, as shown in Fig. S5a and b. For transferring a square pixel (size: 150 μm × 150 μm), a structured stamp (150 μm × 150 μm with 20-μm height) was used in the structured stamping method (Supplementary Fig. 5a). In the intaglio transfer technique, on the other hand, a 150 μm × 150 μm square trench and a flat stamp were utilized (Supplementary Fig. 5b). The QD/stamp interaction and QD/substrate interaction were modelled using the cohesive contact. Surfaces followed the traction-separation behaviour, $t = K\delta$, where t is the traction normal to the surface, δ is the separation between the surfaces, and K is the coefficient representing the bonding strength. If the separation reaches a critical value, the traction gradually degrades to zero, and two surfaces are regarded as debonded. Following values were used in the simulation: $K_{stamp/QD}$ = 4.2×10^{-5} Pa/μm and $K_{substrate/QD}$ = 4.0×10^{-5} Pa/μm in the structured stamping and $K_{stamp/QD}$ = 1.5×10^{-5} Pa/μm and $K_{substrate/QD}$ = 3.0×10^{-5} Pa/μm in the intaglio printing. These values were selected by considering the bonding strength differences. In the structured stamping, $K_{stamp/QD}$ is larger than $K_{substrate/QD}$, but the difference is small. In the intaglio transfer printing, $K_{stamp/QD}$ is smaller than $K_{substrate/QD}$, which is reasonable because of the slow pick-up procedure. The pick-up process was simulated by moving the stamp upward while fixing the substrate. The resulting FEM simulation results for QD transfer-printings are shown in Fig. 2d and e, which are well matched with the experimental results shown in Fig. 2a.

The details of FEM simulations used to analyse the delamination and crack propagation of QD layers near the edge of stamps (Supplementary Fig. 5c and d) are given as follows. The substrate was modelled as a rigid body, whereas the structured stamp and the QD layer were modelled using three-dimensional solid elements. The thickness of the QD layer is ~40 nm, and the corner radius of the elastomeric stamp is ~350 nm. The surfaces followed the traction-separation behaviour, $t = K\delta$. The incompressible Neo-Hookean model was used to represent the polydimethylsiloxane (PDMS) stamp: $W = C_1(I_1 - 3)$, where W is the strain energy

potential, I_1 is the first invariant of the left Cauchy-Green tensor, and C_1 (=383.5 kPa for PDMS) is a material parameter. The QDs were modelled as a plastic material with an initial yield stress of 10^{-4} MPa, and the fracture occurs after the strain reaches 0.01. Figure S5e shows the normal stress distribution to the vertical direction. The stress is localized at the edge of the stamp, initiating the delamination between the stamp and the QD layer (Supplementary Fig. 5e (i)). The delamination proceeds until it meets the delamination between the substrate and the QD layer, which results in the fracture of the QD layer inside of the pixel edge (Supplementary Fig. 5e (ii)). In the intaglio transfer technique, on the other hand, the stress is focused on the edge of pattern, where the crack is formed exactly at that location (Supplementary Fig. 5f (i) and (ii)). The bonding strength between the substrate and the QD layer is much higher than that between the stamp and the QD layer, which explains Supplementary Fig. 5d and f.

Fabrication of mixed white QLEDs (MWQLEDs)

In this work, MWQLEDs refer to QLEDs whose active layer is composed of mixed R, G, and B QDs in the solution. The fabrication process of MWQLEDs is same as that of PWQLEDs, except for the procedure for the active layer. Mixed QD solutions, in different volume ratios of each QD dispersion, were utilized to manufacture the active layer in MWQLEDs; the ratio of each RGB QD dispersion was 1:1:2. Before mixing QD solutions, the concentration of each QD solution was adjusted to make 40-nm-thick QD layers by spin casting. The mixing ratio of the QD solution was matched with the ratio of the RGB pixel area of PWQLEDs (the area ratio of RGB pixels in PWQLEDs is 1:1:2).

Materials for synthesis of colloidal nanocrystals (NCs)

Cadmium oxide (CdO, >99.99%), zinc acetate ($Zn(OAc)_2$, 99.99%), zinc acetate dehydrate ($Zn(OAc)_2 \cdot (H_2O)_2$, 99.9%), lead chloride ($PbCl_2$, 99.999%), elemental sulfur (S, 99.99%), elemental selenium (Se, 99.99%), oleic acid (OA, 90%), 1-octadecen (1-ODE, 90%), trioctylphosphine (TOP, 97%), tributylphosphine (TBP, >93.5%), 1-octanethiol (98.5%), anhydrous cyclohexane, and anhydrous ethanol were purchased from Sigma-Aldrich. Oleylamine (OLAm, 80-90%) was purchased from Acros Organics. Indium iodide (InI_3, 99.999%) and copper iodide (CuI, 99.998%) were purchased from Alfar Aesar. Prior to use, OA, 1-ODE, and OLAm were degassed at 120°C under vacuum for 4 h.

Synthesis of CdSe/CdS/ZnS red QDs

Red light-emitting CdSe/CdS/ZnS core/shell QDs were prepared by multiple injections of precursors. In a glove box, 1.2 mmol of CdO was added into the reaction solvent composed of 1.5 mL of OA and 20.0 mL of 1-ODE. The mixture was degassed under vacuum for 2 h at 130°C and then heated to 300°C under Ar atmosphere. At this elevated temperature, 0.3 mmol of TOPSe (1M) solution was rapidly injected into the Cd-(oleate)$_2$ solution. The reaction was maintained to allow CdSe cores to grow into the desired sizes. Then, 0.9 mmol of 1-octanethiol was slowly introduced into the reacting solution at 300°C to form CdSe/CdS core/shell QDs. After 40 min of CdS shell growth, 4.8 mmol of Zn-(oleate)$_2$ and 4.8 mmol of TBPS solution (2 M) were slowly injected into the solution to form thick ZnS outer shells on the CdSe/CdS QDs. The reaction mixture was kept at 300°C for 15 m for the further growth of the ZnS outer shell. The product was purified by repeating the precipitation/redispersion processes to remove excessive shell reagents. The as-synthesized CdSe/ZnS alloyed QDs were treated with oleic acid to guarantee dispersibility of the QD solution. The FTIR spectrum of QD samples was analysed at ATR-FTR mode on Tensor 27 (Bruker, Germany) installed at the National Center for Inter-university Research Facilities (NCIRF) in Seoul National University.

Synthesis of CdSe/ZnS green QDs

Green light-emitting CdSe/ZnS core/shell QDs were prepared from the reaction between metal-oleate and TOPS and TOPSe. For the preparation of Cd-(oleate)$_2$ and Zn-(oleate)$_2$ solution, 0.2 mmol of CdO and 4.0 mmol of Zn(OAc)$_2$ were added into the reaction solution composed of 6.0 mL of OA and 15.0 mL of 1-ODE in a glove box. The mixture was degassed under vacuum for 2 h at 130°C and then heated to 300°C under Ar atmosphere. At this elevated temperature, 0.2 mmol of TOPSe solution (1M) was rapidly injected into the solution followed by the injection of 4 mmol of TOPS solution (1M) to form ZnS shells on the cores. The reaction mixture was held at 300°C for 15 min. The product was purified by repeating the precipitation/redispersion processes to remove excessive shell reagents.

Synthesis of CdSe/ZnS blue QDs

Blue light-emitting CdSe/ZnS core/shell QDs were prepared using the reaction between metal-oleate and elemental S and Se. To prevent rapid growth of QDs, consequently, in order to obtain blue light-emitting QDs, elemental S and Se were employed as anion precursors

instead of as general precursors such as TOPSe and TOPS solution. For the preparation of Cd-(oleate)$_2$ and Zn-(oleate)$_2$ solution, 1.0 mmol of CdO and 9.0 mmol of Zn(OAc)$_2$ were added into the reaction solution composed of 8.0 mL of OA and 15.0 mL of 1-ODE in a glove box. The mixture was degassed under vacuum for 2 h at 130°C and then heated to 300°C under Ar atmosphere. At 300°C, 1.8 mmol of S and 0.2 mmol of Se dissolved in 3 mL of 1-ODE were injected into the Cd-(oleate)$_2$ and Zn-(oleate)$_2$ solution. After 10 min of reaction, without any purification, 8.0 mmol of TBPS solution (2 M) was slowly injected into the reaction mixture at 300°C. The reaction mixture was held at the same temperature for 50 min for the formation of thick ZnS shells on the cores. The product was purified by repeating the precipitation/redispersion processes to remove excessive shell reagents.

Synthesis of zinc oxide NCs

Zinc oxide (ZnO) NCs were synthesized using a method modified from a previous report[S1]. First, 0.48 g of KOH in 25-ml methanol was added dropwise to 1.23 g of $Zn(OAc)_2 \cdot (H_2O)_2$ in 55-ml methanol over 2 h at 60°C in ambient conditions. This solution is reacted for 2 h at 60°C. The product was purified by repeating the precipitation/redispersion method using the hexane and isopropyl alcohol mixture and redispersed in butanol. Before used as the electron transport layer, the ZnO NC solution was processed through a 200-nm porous cellulose acetate filter.

Synthesis of PbS IV-VI semiconductor QDs

The PbS IV-VI semiconductor QDs were synthesized using the method in our previous report[S2]. In a glove box, 1 mmol of $PbCl_2$ was added into 5 mL of OLAm. The mixture was degassed under vacuum for 2 h at 90°C, forming a clear solution containing metal-amine complex. Then, 0.83 mmol of amine-sulfur complex solution (0.83 mmol elemental sulfur was dissolved in 2.5 mL of oleylamine) was rapidly injected into the Pb-oleylamine complex solution at 90°C. The resulting black colloidal solution was heated to 220°C and held at that temperature for 1 h for further PbS QD growth. The product was purified by repeating the precipitation/redispersion process.

Synthesis of Cu-In-Se I-III-VI semiconductor QDs

The Cu-In-Se I-III-VI semiconductor QDs were synthesized using the method in our previous report[S3]. For the synthesis of 5-nm QDs, 0.5 mmol of copper (I) iodide (0.095 g) and 0.5 mmol of indium (III) iodide (0.248 g) were added into 15 mL of oleylamine. The solution was degassed under vacuum for 30 m at 120°C, forming a clear solution of metal-amine complexes. Oleylammonium selenocarbamate was separately prepared by the reaction of carbon monoxide (CO), 5.0 mmol of selenium powder, and 10 mL of oleylamine at 120°C. The 2.0 mL of oleylammonium selencabamate solution was injected into the metal-amine complex solution at 70°C under an Ar atmosphere. The reaction solution was heated to 210°C and held at that temperature for 20 min. The product was precipitated by ethanol-containing TOP followed by repeating the purification process (precipitation/redispersion method).

Supplementary References

1. Pacholski, C., Kornowski, A. & Weller, H. Self-assembly of ZnO: from nanodots to nanorods. *Angew. Chem., Int. Ed.* **41,** 1188–1191 (2002).
2. Joo, J. *et al.* Generalized and facile synthesis of semiconducting metal sulfide nanocrystals. *J. Am. Chem. Soc.* **125,** 11100–11105 (2003).
3. Yang, J. *et al.* Copper-indium-selenide quantum dot-sensitized solar cells. *Phys. Chem. Chem. Phys.* **15,** 20517–20525 (2013).
